# Nanocell COVID-19 vaccine triggers a novel immune response pathway producing high-affinity antibodies which neutralize all variants of concern

**DOI:** 10.3389/fimmu.2022.1038562

**Published:** 2023-01-27

**Authors:** Steven Y. Gao, Nancy B. Amaro-Mugridge, Jocelyn Madrid-Weiss, Nikolina Petkovic, Natasha Vanegas, Kumar Visvanathan, Bryan R. G. Williams, Jennifer A. MacDiarmid, Himanshu Brahmbhatt

**Affiliations:** ^1^ EngeneIC Pty Ltd., Sydney, NSW, Australia; ^2^ Kumar Visvanathan, Department of Medicine, University of Melbourne, Fitzroy, VIC, Australia; ^3^ Department of Molecular and Translational Science, Hudson Institute of Medical Research, Monash University Faculty of Medicine, Nursing and Health Sciences, Clayton, VIC, Australia

**Keywords:** COVID-19, nanocell vaccine, iNKT activation, memory B cell, memory T cell, variants of concern

## Abstract

Most current anti-viral vaccines elicit a humoral and cellular immune response *via* the pathway of phagocytic cell mediated viral antigen presentation to B and T cell surface receptors. However, this pathway results in reduced ability to neutralize S-protein Receptor Binding Domains (RBDs) from several Variants of Concern (VOC) and the rapid waning of memory B cell response requiring vaccine reformulation to cover dominant VOC S-proteins and multiple boosters. Here we show for the first time in mice and humans, that a bacterially derived, non-living, nanocell (EDV; EnGeneIC Dream Vector) packaged with plasmid expressed SARS-CoV-2 S-protein and α-galactosyl ceramide adjuvant (EDV-COVID-αGC), stimulates an alternate pathway due to dendritic cells (DC) displaying both S-polypeptides and αGC thereby recruiting and activating iNKT cells with release of IFNγ. This triggers DC activation/maturation, activation of follicular helper T cells (T_FH_), cognate help to B cells with secretion of a cytokine milieu promoting B cell maturation, somatic hypermutation in germinal centers to result in high affinity antibodies. Surrogate virus neutralization tests show 90-100% neutralization of ancestral and early VOC in mice and human trial volunteers. EDV-COVID-αGC as a third dose booster neutralized Omicron BA. 4/5. Serum and PBMC analyses reveal long lasting S-specific memory B and T cells. In contrast, control EDVs lacking αGC, did not engage the iNKT/DC pathway resulting in antibody responses unable to neutralize all VOCs and had a reduced B cell memory. The vaccine is lyophilized, stored and transported at room temperature with a shelf-life of over a year.

## Introduction

SARS-CoV-2 (Severe Acute Respiratory Syndrome-Coronavirus type 2) is the causative agent of the COVID-19 pandemic and despite global vaccination efforts the pandemic is failing to abate, particularly with the continued emergence of new variants leading to global waves of breakthrough infections and significant death tolls (1–4). Furthermore, for vaccines to be successful, they need to protect the most vulnerable within our communities, but unfortunately the immune-compromised remain susceptible to SARS-CoV-2 and its ever-evolving variants (5–10).

All current anti-viral vaccines, including COVID vaccines, elicit an antigen-specific antibody response *via* the pathway of antigen presenting cell/B cell receptor antigen recognition and antigen-specific antibody secretion. For decades, these antibodies have been known to be low-affinity antibodies and as a result, even influenza vaccines must be reformulated each year, based on the prevailing mutant strains (11). The same problem has been observed with current COVID vaccines with a race on to make an Omicron-specific vaccine (12).

Scientists are therefore faced with the challenge of producing a vaccine that can better engage parts of the immune system capable of rapidly involving cognate T cell help, leading to B cell somatic hypermutation (SHM) producing antibodies of high and broad affinity with long-lasting memory B cells.

Currently approved COVID vaccines also have logistical issues since they need to be stored and transported at -20^°^C to -70^°^C with a shelf-life of only six to 12 months and several countries having to discard hundreds of millions of doses of outdated wild-type (WA1) homologous vaccine (13).

**Figure 1:**
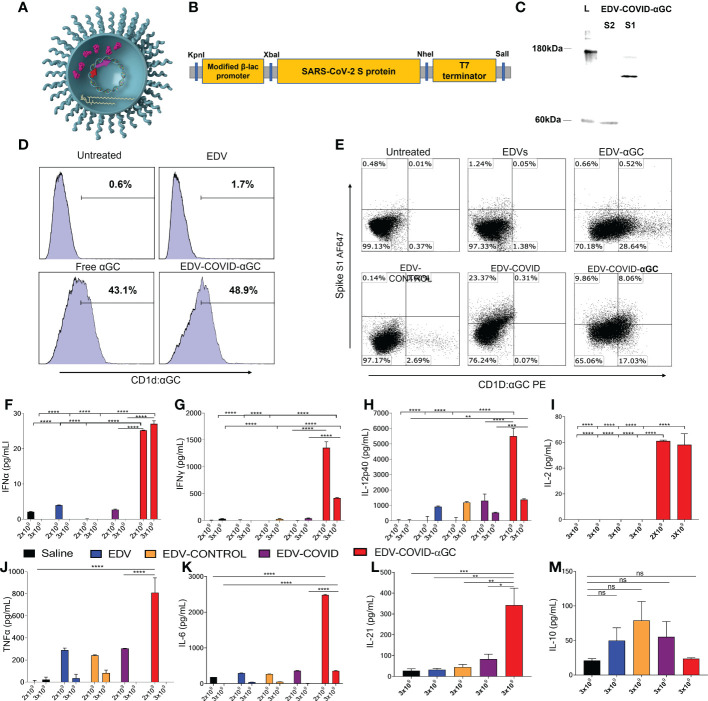
EDV-COVID-αGC formulation, antigen/αGC co-presentation and early cytokine response in mice. **(A)** Image of EDV-COVID-αGC depicting the LPS, membrane and nanocell contents including plac-CoV-2 plasmid, S-protein and αGC. **(B)** Construct: SARS-CoV-2 S-protein nucleotide sequence (Genbank MN908947.3) at the 3′-end of a modified constitutive gene expression β-lactamase promoter and inserted between KpnI 5′ and SalI 3′ sites of the M13 multiple cloning site of pUC57-Kan backbone plasmid to create plac-CoV-2. **(C)** Western blot analysis using MAbs against the S1 and S2 subunit demonstrated the presence of the S-protein within EDV-COVID-αGC. **(D)** FACS analysis showing that EDV-COVID-αGC was able to effectively deliver αGC into murine bone marrow derived, JAWSII, cells and presented through CD1d-ligand to a similar efficiency as free αGC. **(E)** Co-staining of JAWSII cells with anti-CD1d:αGC and anti-spike Abs demonstrating αGC and S-protein delivery by EDVs with EDV-COVID-αGC delivering both S-protein and αGC on the same cell surface. **(F–H)** IFNα, IFNγ, IL-12-p40 levels at 8 h post dose 1 following I.M. injections of female BALB/c mice (n=6) with 2 and 3 (x 10^9^) EDV-COVID-αGC or various controls. **(I)** IL-2 levels in 2 x 10^9^ and 3 x 10^9^ EDV-COVID-αGC particle dose after 8 h post dose 1. **(J, K)** TNFα and IL-6 levels in 2 x 10^9^ and 3 x 10^9^ EDV-COVID-αGC or various controls 8h post dose 1. **(L)** IL-21 levels for 3 x 10^9^ dose measured at day 28. **(M)** IL-10 levels in 3 x 10^9^ EDV-COVID-αGC particle dose after 8 h post dose 1. Units = pg/ml. Asterisks represent significant values (****p ≤ 0.0001; ***p ≤ 0.001; **p ≤ 0.01; *p ≤ 0.05) measured using two-way ANOVA and Tukey’s test or one-way ANOVA and Tukey’s test (IL-2) on GraphPad Prism v 9.4.0. ns, not significant.

Here we describe a novel class of vaccine, designated EDV-COVID-αGC, comprising a 400 nm diameter, non-living, achromosomal nanocell, EDV™ (EnGeneIC Dream Vector) packaged with (i) Type I interferon stimulating bacterial gene expression recombinant plasmid encoding S-protein sequence, (ii) plasmid expressed S-protein produced in the nanocell cytoplasm, and (iii) iNKT cell licensing and type II interferon stimulating glycolipid adjuvant α-galactosyl ceramide (αGC) (**Figure 1A**). EDVs are derived from a mutant non-pathogenic *Salmonella enterica* serovar Typhimurium strain and separate from the parent bacterium in the course of its normal replication due to asymmetric cell division induced by the chromosomal mutation (14, 15). Single chain Fv bispecific (scFv) antibody targeted EDVs have been used to deliver cytotoxic payloads and small molecules to solid cancers in Phase I and IIa clinical trials in several solid tumors. Tumor stabilization/regression, prolonged overall survival, and minimal to no toxicity despite repeat dosing, has been achieved in these end-stage patients who had exhausted all treatment options (16–18).

This work shows that EDV-COVID-αGC can deliver SARS-CoV-2 S-protein and αGC to dendritic cells (DCs) stimulating an S-specific humoral and cellular response with broad-spectrum neutralization against wild-type, Alpha, Beta, Gamma, Delta and Omicron variants at greater than 90% in mice. Furthermore, we present results from six volunteers of the EDV-COVID-αGC Phase I clinical trial which encouragingly echo our pre-clinical data thus far and also demonstrate significant Omicron B.1.1.529, BA.2 and BA.5 neutralization.

## Materials and methods

### Clinical trial ACTRN12621001159842

This is a Phase I/IIa, open label study to determine the safety of EDV nanocells packaged with a plasmid encoding SARS-CoV-2 spike protein in the EDV and a glycolipid α-galactosyl ceramide (EDV-COVID-αGC) in non-COVID-19 infected volunteers, 18 years and older.

All participants in clinical trials signed a patient informed consent form prior to commencement of treatment with approval from St. Vincent’s Hospital Melbourne Human Research Ethics Committee.

Subjects were identified as SARS-CoV-2 naive *via* PCR test and naïve for prior COVID-19 vaccines. History of prior COVID-19 infection was excluded by patient medical history questionnaire. Pre-dose blood samples were taken and assays performed as a baseline level. All subjects received a dose of 9 x 10^9^ EDV-COVID-αGC i.m with an equal booster dose on day 21. All doses were administered in clinic with 3-hour safety monitoring on dosing days, including vital signs, laboratory tests and adverse event monitoring.

Samples were collected at 4 time points: pre-vaccine baseline (day 1), day 21 before the booster vaccination, and day 28 one-week post-boost. Subjects are also scheduled to return for a 2-, 3- and 6-month time point. Each study visit included collection of 20 mL of peripheral blood. The study began in September 2021 and at time of submission there are over 50 volunteers that have come forward to be part of the study. Selected data are presented here for six volunteers.

### Animal studies

Female BALB/c mice, 6-8 weeks old were obtained from the Animal Resource Centre in Western Australia. The mice were acclimatized for one week before the experiments commenced. All experiments were subject to assessment and approval by the EnGeneIC Animal Ethics Committee according to the “Australian code for the use and care of animals for scientific purposes”. Treatment groups (n = 4-10 depending on the experiment) included EDV-COVID-αGC as well as control groups consisting of saline, EDV, EDV-αGC, EDV-CONTROL (Control Plasmid) and EDV-COVID. Initial experiments involved a 2 x 10^9^ i.m. particle dose (in 100 µL injectable saline; 50 µL/flank) into a single flank at day 0, followed by a booster of 1 x 10^9^ (in 50 µL injectable saline) at day 21. Subsequent experiments applied a higher i.m. dose of 3 x 10^9^ particles split into 1.5 x 10^9^ per back flank (in 50 µL injectable saline each) due to limitations of particle volume/concentration acceptable per flank, with a boost of the same dose and mode of delivery at day 21. Choice of dose was based on a pilot study using 2 x 10^9^, 3 x 10^9^, 4 x 10^9^ and 6 x 10^9^ particles i.m. and observation of mouse tolerability including, ruffling and lethargy 24 hours post injection. Mouse data are taken from three experiments, the first two including both dose levels and the last using only 3 x 10^9^. Depending on the experiment, serum and tissues were collected at 8 h, day 7, day 21 and day 28 post-initial injection. Blood was collected *via* heart puncture immediately following euthanasia by anesthetic overdose with Ketamine and Ilium Xylazil-20 (Troy Laboratories, Australia), or tail bleeding for ongoing analysis. Other tissues harvested include spleen, lymph nodes, and bone marrow from the femur.

### Recombinant CoV proteins and antibodies

SARS-CoV-2 spike proteins were purchased from ACRObiosystems Inc. SARS-CoV-2 (Cov-19) S protein, His Tag, super stable trimer (MALS & NS-EM verified) (Cat. #SPN-C52H9) was used in early experiments to analyze IgG and IgM response as well as for Activated Immune Cell Marker assays (AIM). Subsequently, with the emergence of new variants of concern and increased availability of recombinant proteins, the following were purchased: SARS-CoV-2 UK Alpha S1 protein (HV69-70del, Y144del, N501Y, A570D, D614G, P681H, T716I, S982A, D1118H), His Tag (Cat. #SPN-C52H6); SARS-CoV-2 S UK Alpha protein RBD (N501Y), His Tag (Cat. #SPD-C52Hn); SARS-CoV-2 SA Beta S protein (L18F, D80A, D215G, 242-244del, R246I, K417N, E484K, N501Y, D614G, A701V) trimer 50ug Cat. #SPN-C52Hk; SARS-CoV-2 SA Beta S protein RBD (K417N, E484K, N501Y), His Tag (MALS verified) (Cat. #SPD-C52Hp); SARS-CoV-2 Brazil Gamma S1 protein (L18F, T20N, P26S, D138Y, R190S, K417T, E484K, N501Y, D614G, H655Y, T1027I, V11 (Cat. #SPN-C52Hg); SARS-CoV-2 India Delta spike S1 (T95I, G142D, E154K, L452R, E484Q, D614G, P681R), His Tag (Cat. #S1N-C52Ht); SARS-CoV-2 Omicron spike protein HRP (RBD, G339D, S371L, S373P, S375F, K417N, N440K, G446S, S477N, T478K, E484A, Q493R, G496S, Q498R, N501Y, Y505H, His Tag)-HRP (Cat. #Z03730, Genscript); SARS-CoV-2 Omicron spike protein RBD-HRP, BA.2 variant, His Tag (G339D, S371F, S373P, S375F, T376A, D405N, R408S, K417N, N440K, S477N, T478K, E484A, Q493R, Q498R, N501Y, Y505H) (Cat. #Z03741, Genscript). SARS-CoV-2 Omicron spike protein RBD-HRP, BA.4 BA.5 variant (Cat. #CP0007, Genscript).

SARS-CoV-2 (COVID-19) spike antibodies against the S1 and S2 subunits, were purchased from GeneTex (Cat. #GTX135356 and #GTX632604) for western blot confirmation of S protein within EDV™. SARS-CoV-2 (2019-nCoV) spike RBD rabbit PAb, antigen affinity purified (Cat. #40592-T62, Sino Biological) was used for quantitation of the S protein within EDVs using ELISA.

### Cell lines

JAWSII mouse bone marrow derived dendritic cells (ATCC^®^ CRL-11904™) were grown in α-minimum essential medium (MEM; Cat. #M7145, Sigma-Aldrich) with ribonucleosides and deoxyribonucleosides (4 mM L-glutamin, Cat. #G7513, Sigma-Aldrich; 1 mM Sodium Pyruvate, Cat. #11360070, GIBCO; 5 ng/mL GMCSF, Cat. #415-Ml-010, R&D Systems and 20% FBS, Cat. #SFBS-FR, Bovogen) at 37°C, 5% CO_2_.

### Generation of plasmid expressing SARS-CoV-2 S-protein under bacterial promoter

An expression cassette was generated by placing the coding nucleotide sequence for SARS-CoV-2 (Covid-19) S-protein (wild-type sequence; GenBank MN908947.3) on the 3′-end of a modified β-lactamase promoter, which has been previously used for expression in *Salmonella enterica* serovar Typhimurium strains (19). The expression cassette was then inserted between the KpnI 5′ and SalI 3′ sites of the M13 multiple cloning site of pUC57-Kan backbone plasmid to create pLac-CoV2. The sequence was optimised for *S. typhimurium* codon usage before manufacturing by Genscript services. A negative control plasmid, pLac-control, was created as above by removing the CoV2 sequence from the pLac-CoV2 (**Figure 1B**).

### Cloning of pLac-CoV2 and pLac into *Salmonella enterica* serovar Typhimurium EDV producing strain and the assessment of plasmid and S-protein within EDVs

PLac-CoV2 and pLac-CoV2-control were electroporated into a chemically competent *Salmonella enterica* serovar Typhimurium intermediate strain, lacking a plasmid restriction mechanism, using a Gene Pulser Xcell™ (Bio-Rad, Hercules CA) with settings 200-ohm, 25 Hz and 2.5 mV. Transformants were recovered in TSB medium for 1.5 h at 37°C before plating on TSB agar plates containing 75µg/mL kanamycin (Cat. #K4000, Sigma-Aldrich). Isolates were picked into TSB broth with 75 µg/mL kanamycin and plasmid DNA was extracted using the Qiagen miniprep kit as per manufacturer’s instructions (Cat. #27104, Qiagen, Germany). Subsequently, the extracted plasmid DNA from the 4004 strain was electroporated as above into the EnGeneIC Pty. Ltd. EDV producing *Salmonella enterica* serovar Typhimurium strain. Clones containing pLac-CoV2 produce the encoded SARS-COV-2 S-protein, which along with the plasmid DNA, becomes incorporated into EDVs during cell division to produce EDV-COVID. The EDVs containing pLac (EDV-CONTROL) were created in the same way to be used as a negative control.

To determine the plasmid content of EDV-COVID, EDV-COVID-αGC and EDV-CONTROL, plasmids were extracted from 2x10^9^ EDVs using a Qiaprep Spin miniprep kit (Qiagen) following the manufacturer’s instructions. Empty EDV were processed in the same manner as a control. The quantity of DNA plasmids was then measured by absorption at OD_260nm_ using a biophotometer (Eppendorf). The copy number of the plasmids were calculated using the following formula and expressed as copies/particle:


$$Number\;of\;copies=\frac{amount\;\times 6.022\times 10^{23}}{length\;\times 10^{9}\times 660}$$

### Western blot

Proteins from 2 x 10^10^ EDV-COVID were extracted using 100 µL B-PER™ (Bacterial Protein Extraction Reagent; Cat. #89822, ThermoFisher) supplemented with 10% (v/v) lysozyme (Sigma-Aldrich) and 1% (v/v) DNase I (Cat. #EN0521, Qiagen). The extracted samples were then centrifuged at 12000×g for 10 min and the supernatant was collected. The pellet was also collected and resuspended in 100 µL PBS. 23 µL of the supernatant and pellet protein samples were co-incubated with 5 µL of loading buffer and 2 µL DTT (Sigma-Aldrich) at 80°C for 20 min before the entire content of each sample was loaded onto a NuPAGE 4-12% Bis-Tris Mini Protein Gel (Cat. #NP0322BOX, ThermoFisher) and run at 190 V for ~80 min. The gel was then transferred using the iBlot 2 system (ThermoFisher) after which the membrane was blocked using SuperBlock™ blocking buffer (Cat. #37515, ThermoFisher) and subsequently stained with 1:1000 Rabbit poly-clonal anti-SARS-CoV-2 spike (S1) antibody (GeneTex) or 1:1000 mouse monoclonal anti-SARS-CoV-2 spike (S2) antibody (GeneTex) and incubated overnight at 4°C. The membrane was then washed with 1X Phosphate-Buffered Saline, 0.05% Tween^®^ 20 (PBST) and incubated with HRP conjugated anti-rabbit (1:5000) (Cat. #ab199091, Abcam) or anti-mouse (1:5000) (Cat. #31430, ThermoFisher) IgG secondary antibody for 1 h at RT. The blot was developed using Lumi-Light Western Blot substrate (Cat. #12015200001, Roche) and visualized using a Chemidoc MP (Bio-Rad).

### EDV S-protein estimation by ELISA

4 x 10^9^ EDV-COVID particles were pelleted by centrifugation at 13000xg for 8 min. 100 µL of B-Per™ Bacteria lysis agent supplemented with 100 µg/reaction of lysozyme (Cat. #L6876, Sigma) and 5U/reaction rDNase I (Cat. #740963, Macherey-Nagel) was added to each sample and incubated on a vortex shaker for 2 h at 600 rpm at RT. The samples were then mixed with 1:5 Dithiothreitol (Cat. #20290, ThermoFisher) and placed on an 80°C heat block (Eppendorf) at 600 rpm agitation for a further 20 min. Protein quantity was assayed using the DC Protein Assay kit (Cat. #5000111, Bio-rad) following the manufacturer’s specifications.

Standards were generated through serial dilution of the S-protein (ACRObiosystems) to achieve the following concentrations: 2000, 1000, 500, 250, 125, 62.5, 31.3 pg/mL. EDV-COVID S protein samples were diluted 1:1000 in PBS. Standards and EDV S-protein samples, were then coated on the ELISA plate, sealed, and incubated overnight at 4°C. The plates were then washed 3 times with 300 µL PBST using a plate washer. 200 µL protein free blocking buffer (Cat. #786-665, Astral Scientific) was added to the plate which was sealed and incubated at RT for 1 h.

Spike RBD Rabbit PAb detection antibody (Sino Biological) was diluted 1:10000 in 10 mL PBST and 100 µL per well was added and incubated for 1 h at RT. The plate was washed in PBST as above before addition of 100 µL sheep anti-rabbit IgG (H+L)-peroxidase (Cat. #SAB3700920, Merck, 1:10000) in 10 mL PBST. Sealed plates were incubated for 30 min at RT in the dark. The plate was washed again as above and 100 µL of TMB solution (Cat. #34022, ThermoFisher) was added per well. The reaction was stopped by adding 50 µL of 2 M H_2_SO_4_ per well within minutes of TMB addition. The samples were analyzed at OD_450nm_ using a µQuant plate reader (Bio-TEK Instruments, Inc) and KC junior software.

### α-Galactosylceramide loading into EDV-COVID

EDV-COVID nanoparticles carrying the S protein were purified in large batches through bio-fermentation of the parent bacteria *Salmonella enterica* serovar Typhimurium, followed by tangential flow filtration (TFF) to purify the EDV-COVID particles from the parent as previously described (14). EDV-COVID particles were then buffer exchanged from media into PBS pH 7.4 (Dulbecco’s; ThermoFisher) complemented with 0.5% tyloxapol (Cat. #T8761, Sigma-Aldrich) prior to loading with αGC based on a protocol as previously described (20).

Alpha-galactosylceramide glycolipid adjuvant (αGC; Advanced Molecular Technologies, Melbourne) stocks were formulated in 100% DMSO (Sigma). Stock αGC was added to EDV-COVID solutions in PBS at a final concentration of 10 µM (8.58 µg/mL equivalent). Co-incubation of EDV-COVID particles and αGC was performed at 37°C with mixing overnight. Unloaded αGC was removed by washing the particles in PBS pH 7.4 (Dulbecco’s; ThermoFisher) through a 0.2 µm TFF system. EDV-COVID-αGC particles were then concentrated in PBS pH 7.4 followed by buffer exchange to 200 mM Trehalose (Cat. #T9531, Sigma) ready for vial filling and freeze-drying.

EDV-COVID-αGC batch vials underwent quality control testing including particle count, uniformity, sterility, S protein concentration, plasmid copy number and αGC concentration per 10^9^ EDV particles, prior to use in animal experiments and clinical trial. Activity of loaded αGC through dendritic cell (DC) uptake and presentation through the CD1d MHC class I like molecule was carried out as described below.

### αGC uptake and presentation by murine DCs

JAWSII cells (ATCC) were treated with EDV-COVID-αGC in a 96-well Perfecta3D hanging drop plate (Cat. #HDP1385, Sigma-Aldrich) at 1x10^9^ EDV-COVID-αGC per cell. JAWSII cells treated with 2 µg/mL αGC (Advanced Molecular Technologies) served as a positive control. The cultures were then incubated for 24 h at 37°C with 5% CO_2_ and cells were collected and stained with a PE conjugated CD1d: αGC complex antibody (Cat. #12-2019-82, ThermoFisher, 1:2000) and analyzed using a Gallios flow cytometer (Beckman). The results were analyzed using Kaluza Analysis software (V.2.1, Beckman).

### Detection of S-protein and CD1d associated αGC in murine DCs following EDV-COVID-αGC co-incubation

JAWSII cells (ATCC) were seeded onto a 96 well hanging drop plate (Sigma-Aldrich) at 5 x 10^4^ cells/well. EDV only, EDV-αGC, EDV-CONTROL, EDV-COVID and EDV-COVID-αGC were co-incubated with the cells at 1x10^9^ EDVs per well. Untreated JAWSII cells were used as controls. The samples were cultured at 37°C with 5% CO_2_ for 48 h before being collected and co-stained with PE anti-mouse αGC:CD1d complex antibody (ThermoFisher, 1:2000) and SARS-CoV-2 S1 protein polyclonal primary antibody (GeneTex, 1:2000) at room temperature for 30 min in the dark. The samples were then stained with Alexa Fluor 647 goat anti-rabbit IgG (H+L) highly cross-adsorbed secondary antibody (ThermoFisher, 1:1000) at 4°C for a further 20 min and analysed using a Gallios flow cytometer (Beckman Coulter). Mouse IgG2a (Cat. #400214, Biolegend) and rabbit IgG (Abcam) were used as isotype controls. DAPI was used to differentiate live/dead cells and single stained samples were used to generate compensation. The samples were analysed using the Kaluza analysis software (V2.1, Beckman Coulter).

### Extraction of αGC from EDV-COVID-αGC for quantitation

An αGC extraction method was adapted from previous similar studies (21, 22). The necessary number of EDV vials were taken to achieve a total of 1 x 10^10^ EDVs per sample for extraction of αGC. An EDV only sample was used as a negative control.

All lyophilized vials were resuspended in 400 µL of PBS (Dulbecco’s Ca^2+^ Mg^2+^ free, ThermoFisher). Each sample was aliquoted in Eppendorf tubes (i.e., two tubes per sample) and all samples were centrifuged @ 16400xg for 7.5 min. The supernatant was removed from each sample and the EDV pellets were resuspended in 800 µL PBS and centrifuged again as above. The supernatant was removed once again, and all samples were resuspended in 500 µL of UltraPure™ H_2_O (Cat. #10977015, ThermoFisher).

For αGC extraction, each 500 µL sample was transferred to a conical bottom 2 mL microtube (Axygen). One stainless steel bead (5 mm) was added to each sample and samples were then homogenized using agitation on the Qiagen TissueLyser II homogeniser (Qiagen). Homogenisation was carried out in two rounds of 2 min agitations at 25 Hz with a brief stoppage between sets. Lysates were then extracted for lipids by adding 1 mL of chloroform/methanol (2:1 CHCL_3_: MeOH ratio), shaking vigorously by hand and incubating at 37°C for 15 min with sonication every 5 min for 1 min. Following 15 min, samples were centrifuged at 2000xg for 10 min in a benchtop micro centrifuge. The organic layer (bottom) was removed to a fresh tube. The samples were then dried before analysis.

### Quantitative LC-MS/MS analysis of αGC

The standard αGC, and the internal standard (IS), D-galactosyl-ß-1,1’ N-palmitoyl-D-erythro-sphingosine were dissolved in DMSO to either, 1 mM or 2 mM, depending on amount of αGC weighed and size of the vial (both concentrations proved suitable as starting working stocks) with heating at 60-80°C for 5 min if necessary to dissolve. Prior to data acquisition the standard stock solution was used to prepare stock dilutions in MEOH:H_2_0 (95:5). A working IS dilution was prepared with final concentration of 200 ng/mL.

Standards were prepared by using five calibration points of αGC (STD) (62.5, 125, 250, 500, and 1000 ng/mL) spiked with 200 ng/mL IS (22). The standard: IS area ratios were used as calibration curve (CC) points or linearity against which the unknown samples were quantified. The samples were dried and reconstituted in 1ml of working IS dilution.

Samples were acquired along with the freshly prepared CC standards on a TSQ Altis (ThermoFisher) triple quadruple mass spectrometer (MS) interfaced with Vanquish (ThermoFisher) UHPLC (Ultra High-Performance Liquid Chromatography) (LC). The LC-MS instrument method employed for data acquisition was optimised as per Sartorius et al. (2017) (22). Xcalibur and TraceFinder software were used for data acquisition and analysis respectively (ThermoFisher).

The chromatographic analysis (LC) was performed on an Acquity BEH Phenyl column (Waters, 100 × 2.1 mm, 1.7 μm), eluted with a short gradient program from 95:5 MeOH/H2O to 100% MeOH in 1 min followed by an isocratic elution at 100% MeOH for 4 min. Flow rate was set at 0.4 mL/min and column temperature at 40°C. αGC eluted at a RT of 1.53 min, IS at 1.07 min. Two MRM transitions were monitored for both STD and IS for quantitative purposes and to confirm analytical identification. The most intense transitions for each compound (i.e., m/z 856.7 > 178.9 for STD and m/z 698.5 > 89.2 for IS) were used as analytical responses.

### Isolation of serum from mice

Whole blood samples were taken *via* heart puncture following lethal anesthesia, collected into SST vacutainers (Cat. #455092, VACUETTE^®^) and allowed to clot at RT for 1 h. After centrifugation for 10 min at 800xg the serum layer was aliquoted and stored at -80°C for SARS-CoV-2 specific antibody detection by ELISA and neutralizing antibody assays.

### Murine splenocyte isolation

Dissected spleens of treated BALB/c mice were placed in 500 µL of RPMI-1640 medium (Cat. #R8758, Sigma-Aldrich). Spleens were then transferred to a Dounce homogenizer (glass tube and pestle; 7 mL; Wheaton) by emptying the contents directly into the body of the glass homogenizer. 4 mL of RPMI-1640 medium was then added and the plunger used to break down the spleen with 10 passes of the plunger into the homogenizer body. The homogenized tissue was then filtered through sterile 70 µm MACS SmartStrainers (Cat. #130-110-916, Miltenyi Biotec) into 50 mL tubes (Falcon) and the glass homogenizer washed with a further 4 mL and passing this volume through the strainer. Splenocytes were then spun at 350xg for 10 min. and the pellet resuspended in 4 mL RPMI-1640 medium. Red blood cell lysis was then performed by adding 16 mL (1:4 ratio) of Red Cell Lysing Buffer Hybri-Max™ (Cat. #R7757, Sigma-Aldrich) and incubated for 10 min. at room temperature. Cells were further washed as per manufacturer’s recommendations and finally, resuspended in 2.5 mL of autoMACS running buffer (Cat. #130-091-221, Miltenyi Biotec) and passed through a 70 µm MACS SmartStrainer to obtain a single-cell suspension.

### ELISA for measurement of mouse serum cytokines

IFNγ, TNFα, IL-6, IFNα, IL-12p40, IL-10, IL-2 and IL-4 from mouse sera were measured using DuoSet^®^ ELISA kits from R&D Systems (**Table S2**) according to manufacturer’s instructions. Serum levels of IL-21 was analyzed using a LEGEND MAX Mouse IL-21 ELISA kit (Biolegend) following the manufacturer’s instructions. Cytokine concentration was determined by calculating absorbance of the samples against standard curves constructed within the same assay using purified material and expressed as pg/mL (**Figures 1F-M**).

### ELISA for measurement of S-protein RBD and S1 IgG/IgM mouse serum titer

For analysis of anti-RBD specific IgG and IgM antibodies, 96-well plates (Immulon 4 HBX; Thermo Fisher Scientific) were coated at 4°C with 50 µL per well of a 2 µg/mL solution of RBD or S1 protein of the corresponding variant being tested (ACRObiosystems) suspended in PBS (GIBCO). On the following day, the coating protein solution was removed and 100 µL of 3% skim milk blocking solution in PBS/0.1% Tween 20 (PBST) or protein-free blocking solution (G-Biosciences) was added and incubated at RT for 1 h. Serial dilutions of mouse serum were prepared in 1% skim milk/PBST or protein-free blocking solution. The blocking solution was removed and 100 µL of each serum sample was added to the plates and incubated for 2 h at RT. Following incubation, the wells were washed three times with 250 µL of 0.1% PBST, before adding 100 µL of goat anti-mouse IgG (H+L) or IgM (Heavy)– HRP-conjugated secondary antibody (Cat. #31430, #62-6820, ThermoFisher, 1:3000) prepared in 0.1% PBST. The samples were incubated at RT for 1 h and washed three times with 0.1% PBST. Once completely dry, the samples were visualized by incubating with TMB for 15 min. The reactions were then terminated, and the samples were read at OD_450nm_ using a KC Junior plate reader (BioTek Instruments).

Antibody titer was determined using ELISA by generating eight 1:3 serial dilutions of the treated mouse serum samples and is expressed as the endpoint titer (reciprocal of the highest serum dilution giving an OD above the cut-off set at two standard deviations above mean negative control (PBST) reading.

### Murine B cell extraction from bone marrow

0.5 mL microfuge tubes were punctured at the base with a 21-gauge needle and placed inside a 2 mL tube. BALB/c mice were euthanized with CO_2_ and placed onto a sterile surgical pad in a class II biosafety cabinet. The mouse abdominal area and hindlimb skin were sanitized with 70% ethanol swabs. Using blunt-end sterile scissors, the surface muscles were dissected to locate the pelvic-hip joint. The hind leg was dissected from the pelvic-hip joint with sharp sterile scissors. The tibia was separated from the hind leg below the knee joint.

Isolated murine tibia and femur were placed in the 1 mL tubes with the cut side of the bone at the bottom. Bone marrow cells were extracted from the tibia and femur *via* 30 s centrifugation at 10000xg. Pelleted cells were resuspended in 1 mL RPMI-1640 medium (Cat. #R8758, Sigma-Aldrich) and incubated with Hybri-Max™ Red Cell Lysing Buffer (Cat. #R7757, Sigma-Aldrich) for 5 min. The lysis buffer was neutralized with 15 mL of RPMI-1640 medium supplemented with 10% Fetal Bovine Serum (FBS) (Cat. #554656; BD) and centrifuged at 300xg for 10 min. Cells were resuspended in a final volume of 10 mL of RPMI-1640 medium for final counting. B cells were isolated using the Pan B Cell Isolation Kit (Cat. #130-095-813, Miltenyi Biotec) as per manufacturers’ instructions.

### Murine B cell stimulation and ELISA

ELISA micro plates were coated with 2 µg/mL SARS-CoV-2 S-protein trimer (ACRObiosystems) and incubated overnight at 4°C. Microplates were washed 3x with phosphate-buffered saline (PBS) and blocked with 200 µL/well of Protein-Free Blocking Buffer PBST (Cat. #786-665, G-Biosciences) for 2 h at RT.

Mouse bone marrow derived B cells were isolated from treated mice and 1x10^5^ cells were seeded into each well in 200 µL AIMV media and incubated at 37°C for 48 h.

At the end of the incubation period, the cells were removed from each well and each microplate was washed 5x with 200 µL/well of 0.05% Tween 20 in PBST. The samples were then incubated in 100 µL/well of 1:5000 mouse IgG-HRP in PBST for 2 h at RT in the dark before washing 3x in 250 µL PBST. The presence of spike specific IgG was detected by adding 100 µL/well of TMB Substrate System and allowed to incubate for 10 min, by which time a color solution formed, and no longer than 20 min. Enzyme reaction was stopped by adding 50 µL/well of 2N H2SO4 Stop Solution. The samples were analyzed using a CLARIOstar microplate reader (BMG LABTECH) at OD_450nm_ with OD_540nm_ as the reference wavelength and analyzed using the MARS software (BMG LABTECH).

### Activation-induced markers assay on murine splenocytes

Isolated splenocytes were seeded at 1 x 10^6^ cells/200 µL/well in AIMV (Life Technologies) serum free media in a 96-well U-bottom plate. Cells were stimulated with 1 µg/mL SARS-CoV-2 trimer (ACRObiosystems) for 24 h at 37°C, 5% CO_2_. 1 µg/mL DMSO was used as a negative control and 10 µg/mL PHA (Cat. #L2769-2MG, Sigma) as a positive control. After 24 h of stimulation, samples were collected in 1.5 mL microfuge tubes by pipetting up and down to collect the cells and centrifuged at 300xg for 10 min. The supernatant was collected and frozen for processing for IFNγ by ELISA (DuoSet, R&D Systems).

For T cells activation staining the cell pellets from above were washed twice in 500 µL FACS buffer (MACS buffer; Miltenyi), centrifuging as above. Final cell pellets were resuspended in 500 µL FACS buffer and stained with the appropriate antibody (included in the kit) for 30 min at RT in the dark (**Table S3**). Cells were then centrifuged at 300xg for 5 min and washed twice with 500 µL FACS buffer. Cells were then fixed in 1% paraformaldehyde for 10 min at 4°C and after that centrifuged at 300xg for 5 min again. Final resuspension was in 300 µL of FACS buffer before analyzing on a Gallios flow cytometer (Beckman). Single stain samples and mouse IgG isotype controls were used to create compensation for the staining.

### Th1/Th2 phenotyping on murine splenocytes

Th1/Th2 phenotyping was carried using the Mouse Th1/Th2/Th17 phenotyping kit (Cat. #560758, BD). Firstly, as per AIM assay, isolated splenocytes were seeded at 1 x 10^6^ cells/200 µL/well in AIMV (Life Technologies) serum free media in a 96-well U-bottom plate. Cells were stimulated with 1 µg/mL SARS-CoV-2 trimer (ACRObiosystems) for 24 h at 37°C, 5% CO_2_. 1 µg/mL DMSO was used as a negative control and 10 µg/mL PHA (Cat. #L2769-2MG, Sigma) as a positive control. After 24 h of stimulation, 1 μL of BD GolgiStop™ (protein transport inhibitor, Cat. #51-2092KZ, BD) per 200 µL of cell culture was added, mixed thoroughly, and incubated for a further 2 h at 37°C. Cells were then centrifuged at 250xg for 10 min and washed 2 times with the stain buffer supplied in the kit (“FBS”). The cells were counted and approximately 1 million cells were transferred to each flow test tube for immunofluorescent staining as per manufacturer’s instructions. Cells were protected from light throughout the staining procedure. Firstly, cells were fixed by spinning at 250xg for 10 min at RT and thoroughly resuspending in 1 mL of cold BD Cytofix™ buffer (provided in the kit or Cat #554655, BD) and incubated for 10-20 min at RT. Following fixation cells were pelleted at 250xg for 10 min at RT and washed twice at RT in stain buffer (FBS). The stain buffer was removed by spinning and the cell pellet was resuspended in 1X BD Perm/Wash™ buffer (Cat. #554723, BD) diluted in distilled water, and incubated at RT for 15 min. Cells were spun down at 250xg for 10 min at RT and the supernatant removed. For staining, the fixed/permeabilized cells were thoroughly resuspended in 50 μL of BD Perm/Wash™ buffer incubated with 20 µL/tube of cocktail included in the kit (Mouse CD4 PerCP-Cy5.5 (clone: RM4-5), Mouse IL-17A PE (clone: TC11-18H10.1), Mouse IFN-GMA FITC (clone: XMG1.2), Mouse IL-4 APC (clone: 11B11) or appropriate negative control. Samples were incubated at RT for 30 min in the dark before proceeding to FACS analysis on a Gallios flow cytometer (Beckman). Compensation was performed manually for each channel using single stained controls.

### SARS-CoV-2 surrogate virus neutralization test (mouse and human samples)

Assessment of neutralizing antibodies was carried out using the FDA approved “cPASS SARS-CoV-2 Surrogate Virus Neutralization Test RUO Kit” (Cat. #L00847-A, Genscript) (23). The kit is a blocking ELISA detection tool mimicking the virus neutralization process, suitable for use with serum from mice and other species. The capture plate is precoated with hACE2 protein. The necessary number of hACE2 coated plate strips were placed on the plate and the remainder stored at 2-8°C. HRP-RBD (wild-type, Genscript) was diluted 1:1000 in HRP dilution buffer provided to a total of 10 mL as per protocol. Mouse and human serum samples, PBMC supernatant and positive and negative controls were diluted 1:10 (10 µL + 90 µL sample dilution buffer) and pre-incubated with HRP-RBD in a 1:1 ratio (60 µL + 60 µL) to allow binding of neutralizing Abs with HRP-RBD. Mixtures were incubated at 37°C for 30 min. Samples or controls were added to the appropriate wells at 100 µL/well. The plate was covered with plate sealer and incubated at 37°C for 15 min. The sealer was then removed and the plate washed 4 times with 260 µL of 1X wash solution. The plate was pat dried after washing. 100 µL of TMB solution was then added to each well and the plate incubated in the dark at RT for up to 15 min. 50 µL of stop solution was added to terminate the reactions. Absorbance was analyzed at OD_450nm_ immediately using a CLARIOstar microplate reader. HACE2 receptor binding inhibition was calculated using the formula provided by the manufacturer (% inhibition=(1-OD value of sample/OD value of negative control) x 100%. As per spec sheet a positive value was interpreted as > 30% and a negative as < 30%.

For the assessment of neutralizing antibodies against variant SARS-CoV-2 strains the following HRP-RBD proteins were purchased from Genscript for substitution into the cPASS kit: SARS-CoV-2 Alpha S-protein (RBD, E484K, K417N, N501Y, Avi & His tag)-HRP (Cat. #Z03596), SARS-CoV-2 Beta S-protein (RBD, N501Y, Avi & His tag)-HRP (Cat. #Z03595), SARS-CoV-2 Gamma S-protein (RBD, E484K, K417T, N501Y, Avi & His Tag)-HRP (Cat. #Z03601), SARS-CoV-2 Delta S-protein (RBD, L452R, T478K, Avi & His Tag)-HRP (Cat. #Z03614), SARS-CoV-2 Omicron S-protein HRP (RBD, G339D, S371L, S373P, S375F, K417N, N440K, G446S, S477N, T478K, E484A, Q493R, G496S, Q498R, N501Y, Y505H, His Tag)-HRP (Cat. #Z03730). SARS-CoV-2 Omicron BA.2 S-protein HRP (RBD, His Tag) (Cat. #Z03741), SARS-CoV-2 Omicron BA.4/BA.5 S-protein HRP (RBD, His Tag) (Cat. #CP0007).

### FACS analysis of T cells and B cells in human samples

T cell analysis was conducted using DuraClone IM T cell subsets tube (Cat. #B53328, Beckman Coulter). 1 x 10^6^ purified PBMCs were added to the tubes directly in 100µl and incubated at RT for 30 min in the dark. The samples were then pelleted at 300xg for 5min and washed once in 3 mL of PBS. The final samples were resuspended in 500 µl of PBS with 0.1% formaldehyde. Compensation for the assay was generated using the Compensation Kit provided in the IM DuraClone T cell subset tube using purified PBMCs.

B cell analysis was conducted using volunteer PBMCs using SARS-CoV-2 S-B Cell Analysis Kit, human (Miltenyi Biotec, 130-128-022). In short, PBMCs were stained with SARS-CoV-2 S-protein-Biotin was then then co-labelled with Streptavidin PE and Streptavidin PE-Vio 770 to eliminative the chance of non-specific binding. Cells were then stained with 7AAD, CD19, CD27, IgG, and IgM before analysed using FACS. All compensations were conducted using UltraComp eBeads™ Plus Compensation Beads (Cat. #01-3333-42, ThermoFisher). Samples were analysed using a Gallios flow cytometer (Beckman Coulter) and analysed using the Kaluza software (Beckman Coulter).

Samples were processed using a Gallios flow cytometer (Beckman) and the results were analyzed using the Kaluza Analysis software (ver 2.1, Beckman).

### Activation-induced markers assay in human samples

Volunteer PBMCs were seeded at 1 x 10^6^ cells/200 µL/well in AIMV (Life Technologies) serum free media in a 96-well U-bottom plate. Cells were stimulated with 2 µg/mL SARS-CoV-2 trimer (ACRObiosystems) for 24 h at 37°C, 5% CO_2_. 2 µg/mL DMSO was used as a negative control and PHA (Cat. #00-4977-93, eBiosciences) as a positive control. After 24 h of stimulation, samples were collected in 1.5 mL microfuge tubes by pipetting up and down to collect the cells and centrifuged at 300xg for 10 min. The supernatant was collected and frozen for processing for IFNγ by ELISA (DuoSet, R&D Systems) and for SARS-CoV-2 wild-type surrogate virus neutralization test using the cPASS kit (Genscript). The negative controls of the samples were also used for IL-21 analysis using IL-21 Human ELISA kit (Cat. #BMS2043, ThermoFisher) following the manufacturer’s instructions.

### Statistical analysis

For mouse cytokine studies, Statistical significance was calculated using one way (IL-2) and two way ANOVA (IFNα, IFNγ, TNFα, IL-6) and Tukey’s test and ANOVA with Kruskal-Wallis test (IL-21, IL-10). For IgG and IgM antibody titres in mouse studies, *P* values were calculated using two way ANOVA and Tukey’s test and Kruskal-Wallis test. For murine B cell stimulation and ELISA analysis, Statistical comparison of EDV-COVID-αGC treated groups (2 x 10^9^ and 3 x 10^9^) with the respective saline control was performed using one way ANOVA. For mouse AIM assays, statistical comparison of EDV-COVID-αGC treated groups (2 x 10^9^ and 3 x 10^9^) with the respective saline control was performed using one way ANOVA. For human trial results, *P* values were calculated using ANOVA or paired t test. LOWESS curve was used for B cell analysis from volunteer PBMCs. The level of significance is expressed as follows: **** *p*≤ 0.0001; *** *p*≤ 0.001; ** *p*≤ 0.01; * *p*≤ 0.05. All statistical analyses were performed on GraphPad Prism v 9.0.4.

### Data and materials availability

All data needed to evaluate the conclusions in the paper are present in the paper or the supplementary materials. This study did not generate any unique datasets or code.

## Results

### EDV-COVID-αGC vaccine carrying spike antigen plus iNKT stimulating α-galactosylceramide

In this study, we created EDV-COVID-αGC, a dual packaged nanocell carrying both the SARS-CoV-2 spike protein and the glycolipid adjuvant, α-galactosylceramide (**Figure 1A**). The pLac-CoV2 bacterial recombinant plasmid expressing SARS-CoV-2 S-protein under a modified β-lactamase promoter (**Figure 1B**), was transformed into the EDV producing *S. typhimurium* and purified EDV-COVID nanocells were shown to contain both subunits of the S-protein by western blot using a polyclonal antibody against S1 and a monoclonal antibody against the S2 subunit (**Figure 1C**). EDV plasmid extraction and quantitation gave a plasmid copy number of ~100 copies pLac-CoV2 per EDV (**Table S1**) whilst protein quantitation showed ~16 ng of spike protein per 10^9^ EDVs (**Figure S1**).

Purified EDV-COVID were loaded with αGC to produce EDV-COVID-αGC and LC-MS/MS measurement from lipid-extracted EDV-COVID-αGC showed ~60 ng of αGC per 10^9^ EDVs (**Figure S2**). Flow cytometric analysis of murine JAWS II cells treated with EDV-COVID-αGC and stained with anti-CD1d:αGC demonstrated the uptake and CD1d-mediated surface presentation of αGC (**Figure 1D**). Furthermore, co-staining of JAWS II cells with anti-spike S1 and anti-CD1d:αGC, confirmed the presentation of both S-protein and αGC on the surface of DCs following co-incubation with EDV-COVID-αGC (**Figure 1E**).

### Early cytokine response and iNKT engagement in mice treated with EDV-COVID-αGC

Intramuscular (i.m.) inoculation of BALB/c mice with a single first dose of 2 x 10^9^ or 3 x 10^9^ EDV-COVID-αGC resulted in 8 h serum samples showing elevated Th1 cellular immune response cytokines compared to controls. As shown in (**Figures 1F, G**), IFNα and IFNγ rose to significantly higher levels (*p* ≤ 0.0001) in EDV-COVID-αGC groups for both dose levels compared to the respective dose controls including Saline, EDV, EDV-CONTROL (spike-negative plasmid) and EDV-COVID (spike protein alone). IL-2 rose significantly in the 3 x 10^9^ dose EDV-COVID-αGC treatment but was not detected in any of the 3 x 10^9^ controls (*p* ≤ 0.0001) (**Figure 1I**). 2 x 10^9^ EDV-COVID-αGC treatment was comparable to the 3 x 10^9^ dose (**Figure 1I**). IL-12p40 rose significantly (*p* ≤ 0.0001) in the EVD-COVID-αGC group at 2 x 10^9^ compared to all controls and significantly compared to saline (*p* ≤ 0.0001) and EDV-COVID (*p* ≤ 0.001) at 3 x 10^9^ (**Figure 1H**). The significant difference between EDV-COVID-αGC and EDV-COVID for all cytokines demonstrates the impact of adding αGC and correlates to iNKT pathway engagement. 2 x 10^9^ dose level for TNFα showed a significant increase compared to saline and EDV-COVID (*p* ≤ 0.0001) but not at the 3 x 10^9^ dose (**Figure 1J**). The observed reduced response in cytokine levels for 3 x 10^9^ dose remains to be further tested to determine whether there is a timing issue associated with increase particle amount. IL-21, a Th2 cytokine crucial in antiviral activity was significantly elevated in mice treated with EDV-COVID-αGC by 28 days compared to saline (*p* ≤ 0.001) and all other controls (*p* ≤ 0.01) (**Figure 1L**). IL-10, is a Th2 inflammatory cytokine which was not elevated significantly (ns) in EDV-COVID-αGC, EDV, EDV-CONTROL (spike-negative plasmid) and EDV-COVID (spike protein alone) groups compared to saline (**Figure 1M**).

TNFα and IL-10, part of the innate immune response to the EDV associated LPS, occurred to a similar extent in all the EDV containing groups (**Figures 1J, M**). IL-6 was shown to be elevated in the EDV-COVID-αGC injected group compared to all the controls at both dose levels (*p* ≤ 0.0001) (**Figure 1K**). The only observable side effect exhibited by mice due to the secretion of TNFα and IL-6 was slight ruffling which resolved within 16-24 h (**Figures 1J, K**).

### Production of S-specific IgG and IgM antibody titers in mice

**Figure 2:**
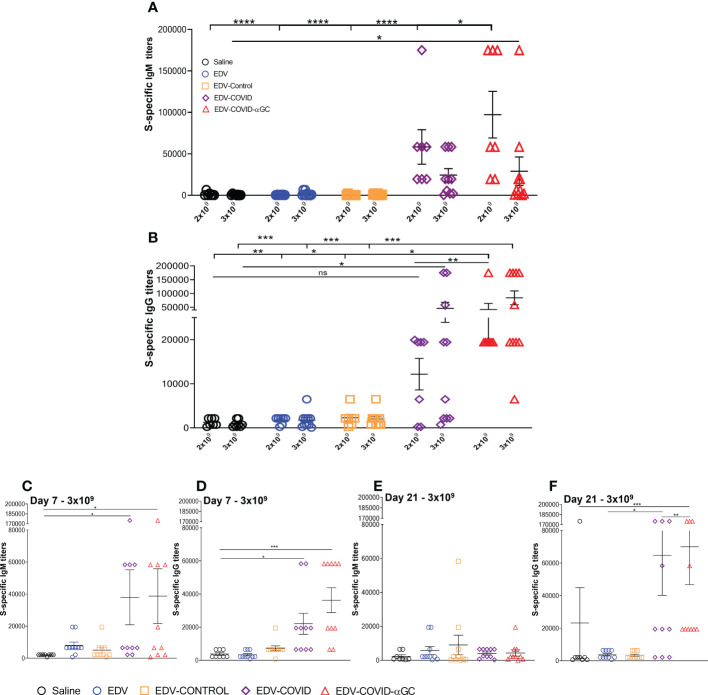
S-specific IgM and IgG titers in female Balb/c mice treated with EDV-COVID-αGC on day 1 and boosted on day 21. **(A)** Day 28 IgM S-protein specific titers for 2 x 10^9^ (n = 7) and 3 x 10^9^ dose (n = 10) levels. **(B)** Day 28 IgG S-protein specific titers for 2 x 10^9^ (n = 7) and 3 x 10^9^ (n = 10) dose levels. **(C–F)** IgM and IgG S-protein specific titers for 3 x 10^9^ (n = 10) dose at day 7 **(C, D)** and at day 21 (n = 10) **(E, F)**. Asterisks representing significant values (****p ≤ 0.0001; ***p ≤ 0.001; **p ≤ 0.01; *p ≤ 0.05) and numerical *p* values were measured using two way ANOVA and Tukey’s test or one-way ANOVA and Tukey’s test (2 D and F) on GraphPad Prism v 9.4.0.

Mice dosed i.m. with 2 x 10^9^ or 3 x 10^9^ EDVs and an equal boost at day 21 were analyzed for serum IgM and IgG antibody titers at day 28 using S-protein-specific ELISA. EDV-COVID-αGC gave significantly elevated IgM at the 2 x 10^9^ dose (**Figure 2A**) and compared to Saline, EDV, EDV-CONTROL (*p ≤ *0.0001) and EDV-COVID (*p* ≤ 0.05) groups. Antibody titers were higher for EDV-COVID-αGC compared to EDV-COVID. IgM levels were significantly elevated compared to saline controls by day 7 for both EDV-COVID and EDV-COVID-αGC (*p* ≤ 0.05) (**Figure 2C**). By day 21 prior to boosting, IgM titers dropped for both EDV-COVID and EDV-COVID-αGC groups (**Figure 2E**).

S-specific mouse serum IgG titer levels rose significantly in the EDV-COVID-αGC group compared to saline (*p* ≤ 0.01), EDV, EDV-CONTROL (*p* ≤ 0.05) and EDV-COVID (*p* ≤ 0.01) controls at 2 x 10^9^ (**Figure 2B**). The EDV-COVID group at 2 x 10^9^ gave titers that were not significantly different to saline. IgG titers in the 3 x 10^9^ EDV-COVID-αGC group were significantly different compared to saline, EDV and EDV-CONTROL (*p* ≤ 0.001). Though not significantly different to EDV-COVID at this dose, the EDV-COVID group showed lower overall significance compared to saline (*p* ≤ 0.05). S-specific IgG titers which rose by 7 days for EDV-COVID and EDV-COVID-αGC (*p* ≤ 0.05 and 0.001 respectively) (**Figure 2D**), remained elevated by day 21 for IgG, particularly in the EDV-COVID-αGC group vs EDV-COVID (*p* ≤ 0.001 vs 0.05) (**Figure 2F**).

### S-protein-specific B and T cell response in mice treated with EDV-COVID-αGC

**Figure 3:**
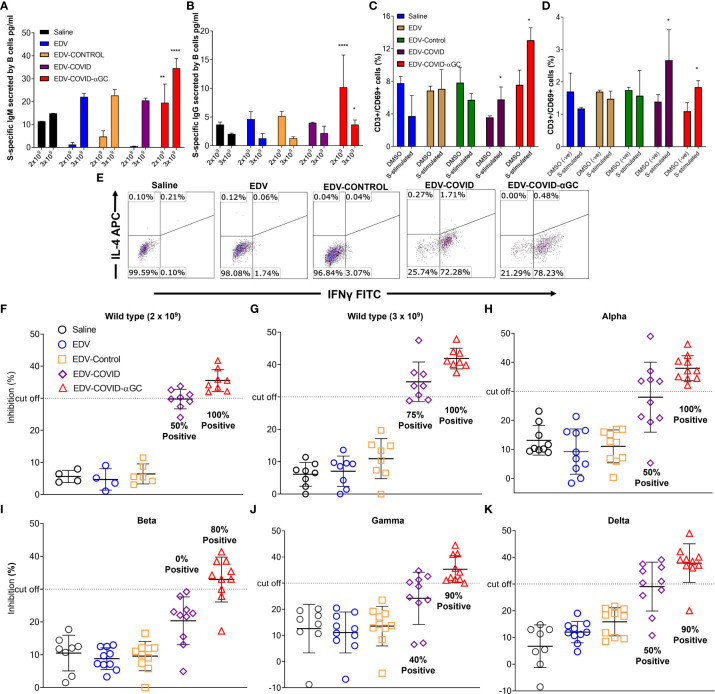
S-protein-specific B and T cell response in murine bone marrow derived B cells and splenocytes and Surrogate Viral Neutralization Test (sVNT) on mouse serum from female Balb/c mice dosed on day 1 and boosted on day 21. S-protein specific IgG and IgM production from bone marrow derived B cells isolated from 2 x 10^9^ and 3 x 10^9^ treated mice (n = 6) at day 28 post-initial dose following *ex-vivo* stimulation with SARS-CoV-2 S-protein. **(A)** IgM (pg/ml) concentrations in 2 x 10^9^ dose groups and 3 x 10^9^ dose groups. **(B)** IgG (pg/ml) concentrations in 2 x 10^9^ dose groups and 3 x 10^9^ dose groups. **(C)** Change in CD69 expression within the CD8^+^ cytotoxic T cell population following the stimulation of *ex vivo* splenocytes with wild type SARS-CoV-2 S-protein in the 2 x 10^9^ dose groups and **(D)** 3 x 10^9^ dose groups compared to DMSO (negative) stimulated controls. Data presented as mean ± SEM. Asterisks represent significant values (****p ≤ 0.0001; **p ≤ 0.01; *p ≤ 0.05) calculated using one way ANOVA on GraphPad Prism v 9.4.0. **(E)** IFNγ (Th1) and IL-4 (Th2) expression within the CD3^+^ CD4^+^ T cell population in SARS-CoV-2 S-protein stimulated *ex vivo* splenocytes. **(F–K)** Viral neutralization tests (VNTs) using the cPASS™ SARS-CoV-2 Neutralizing Antibody Assay (FDA approved) for detection in various species was used to assess inhibition of RBD binding to hACE2 receptor. **(F)** VNTs using the serum of mice immunized with 2 x 10^9^ (n = 4, 8) and **(G)** 3 x 10^9^ (n = 8) EDVs against SARS-CoV-2 RBD wild-type. Subsequent VNTs were conducted using the serum of 3 x 10^9^ EDV immunized mice against the Alpha **(H)**, Beta **(I)**, Gamma **(J)** and Delta **(K)** variant RBDs (n = 8). Dotted line represents sVNT 30% cut-off correlating with a positive PRNT90 for 1:10 dilution of sera.

To study the B cell response after immunization of mice at both 2 x 10^9^ and 3 x 10^9^ levels, bone marrow derived B cells were stimulated *ex-vivo* with SARS-CoV-2 S-protein and B cell secreted S-specific IgM and IgG titers were measured. A dose of 2 x 10^9^ EDV-COVID-αGC resulted in significantly elevated IgM and IgG levels (*p* ≤ 0.01, *p* ≤ 0.0001 respectively) compared to all other groups dosed at 2 x 10^9^ and similarly, 3 x 10^9^ EDV-COVID-αGC resulted in significantly elevated IgM and IgG levels compared to all other groups at 3 x 10^9^ (*p* ≤ 0.0001, *p* ≤ 0.05 respectively) (**Figures 3A, B**).

When analyzing the early T cell activation marker CD69 as a percentage of CD3 within the *ex vivo* splenic CD8^+^ T cell population by flow cytometry, a dose of 2 x 10^9^ EDV-COVID-αGC gave a higher T cell response (*p* ≤ 0.05) following stimulation with S-protein compared to DMSO stimulation (*p* ≤ 0.05) (**Figure 3C**). This higher CD69^+^/CD3^+^ ratio was also observed at a dose of 3 x 10^9^ (*p *= 0.0185) for S-protein stimulation compared to DMSO (*p* ≤ 0.05) (**Figure 3D**).

In **Figure 3E**, Th1/Th2 phenotyping studies following S-protein stimulation of *ex-vivo* splenocytes, show that CD4^+^ T cells from EDV-COVID and EDV-COVID-αGC mice produced IFNγ but not IL-4 within 24 h compared to other groups, which had no response.

### Surrogate virus RBD neutralization of VOC by EDV-COVID-αGC

FDA approved cPASS™ sVNT kit was used to evaluate the level of neutralizing antibodies in mouse serum at day 28 post i.m. inoculation of 2 x 10^9^ and 3 x 10^9^ (for wild type strain) and 3 x 10^9^ for Alpha, Beta, Gamma and Delta strains. According to the kit’s specifications a sample is deemed positive for PRNT_90_ at 30% level of inhibition (23, 24). Following these guidelines, at a dose of both 2 x 10^9^ and 3 x 10^9^, 100% of the mice treated with EDV-COVID-αGC neutralized RBD from wild type SARS-CoV-2, while 50% and 75% respectively for the corresponding doses of EDV-COVID showed RBD neutralization (**Figures 3F, G**). 100% of mice treated with 3 x 10^9^ EDV-COVID-αGC neutralized the respective RBDs from Alpha strain, 80% Beta and 90% Gamma and Delta, however vaccination using EDV-COVID without αGC yielded a noticeably poorer response (**Figures 3H–K**).

### EDV-COVID-αGC clinical trial

**Figure 4:**
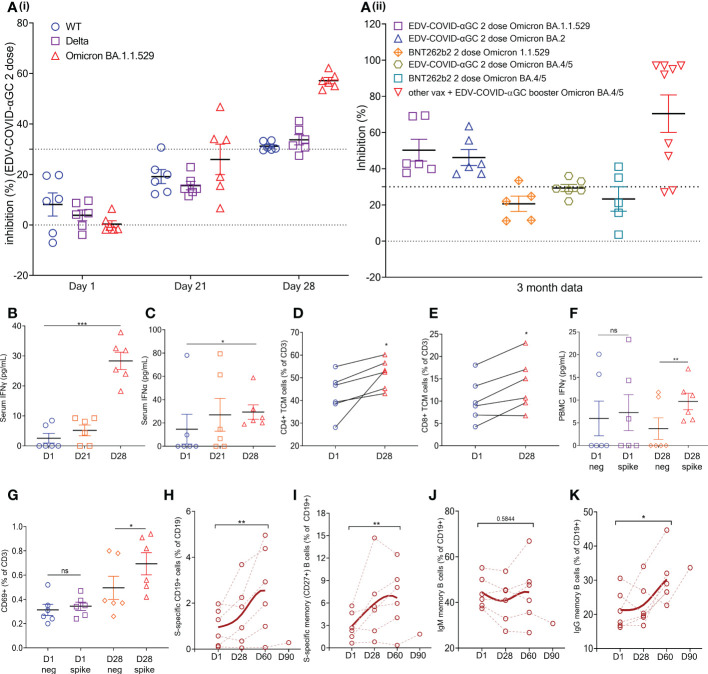
EDV-COVID-αGC Clinical trial: Data from the 6 volunteers. **(Ai)** SVNT analysis of volunteer serum on day 1, 21, 28 post-initial injection and a booster on day 21 against wild type, Delta and Omicron B.1.1.529 variants of the SARS-CoV-2 RBD. **(Aii)** SVNT analysis of volunteer serum against Omicron B.1.1.529 and Omicron BA. 2 and BA. 4/5 variants of the SARS-CoV-2 RBD, at 3 months post 2 doses of EDV-COVID-αGC only, 2 doses of BNT262b2 only, and EDV-COVID-αGC as a booster. Dotted line represents sVNT 30% cut-off correlating to a positive PRNT90 for 1:10 dilution of sera. **(B, C)** Serum IFNγ and IFNα levels respectively on day 1, 21 and 28 after initial injection and a day 21 booster. **(C)** Serum IFNα levels (pg/ml) on day 1, 21 and 28 post-initial injection. **(D)** CD4^+^ central memory T cells (TCM) (CD45RA^-^ CD27^+^ CCR7^+^ CD3^+^ CD4^+^) analysis on day 1 and day 28. **(E)** CD8^+^ central memory T cells (CD45RA^-^ CD27^+^ CCR7^+^ CD3^+^ CD8^+^) analysis on day 1 and day 28. **(F)** *Ex vivo* PBMC production of IFNγ following SARS-CoV-2 S-protein stimulation on day 1 and day 28. **(G)** CD69 expression in T cells (CD45^+^ CD3^+^ CD69^+^) in *ex vivo* PBMCs following SARS-CoV-2 S-protein stimulation on day 1 and day 28. **(H)** Amount of S-protein specific CD19^+^ B cells in PBMCs on day 1, 28, 2 months and 3 months following initial injection. **(I)** Amount of S-protein specific CD19^+^ CD27^+^ memory B cells in PBMCs on day 1, 28, 2 months and 3 months following initial injection. **(J)** Amount of IgM^+^ CD19^+^ CD27^+^ memory B cells in PBMCs on day 1, 28, 2 months and 3 months following initial injection. **(K)** Amount of IgG^+^ CD19^+^ CD27^+^ memory B cells in PBMCs on day 1, 28, 2 months and 3 months following initial injection. Data presented as mean ± SEM, ns, not significant. Asterisks represent significant values (***p ≤ 0.001; **p ≤ 0.01; *p ≤ 0.05) between stated groups and calculated using paired t test on GraphPad Prism v 9.4.0.

Healthy volunteers receiving 9 x 10^9^ EDV-COVID-αGC from our phase I study cohort exhibited strong neutralizing activity (>PRNT_90_ equivalent) using the cPASS SARS-CoV-2 Surrogate Virus Neutralization Test Kit, against wild-type, Delta and Omicron B.1.1.529 as seen at day 28 (**Figure 4Ai**). The level of Omicron B.1.1.529 neutralization was sustained for at least 3 months, at which time point newly emergent Omicron variants BA.2 and BA. 4/5 were also tested (**Figure 4Aii**). For comparison, we also included neutralization results for Omicron B.1.1.529 and BA. 4/5 from 5 volunteers who had received 2 doses of BNT262b2 at 3-4 months previously (**Figure 4Aii**). In addition, we found the neutralization level against BA. 4/5 increased significantly in volunteers who had previously received other available vaccines and were then boosted with EDV-COVID-αGC at least 4 months later (**Figure 4Aii**).

Serum IFNγ (**Figure 4B**) and IFNα (**Figure 4C**) levels where increased and remained elevated in EDV-COVID-αGC trial volunteers (*p* ≤ 0.001 and *p* ≤ 0.05 respectively) compared to day 1 baseline). Similar to the mouse splenocyte data (**Figure 3E**), *ex vivo* PBMCs isolated on day 28 responded to SARS-CoV-2 S-protein stimulation and produced elevated levels of IFNγ (*p* ≤ 0.01) (**Figure 4F**). Total PBMC analysis showed that there is an increase in CD4^+^ (CD45RA^-^ CD27^+^ CCR7^+^ CD3^+^ CD4^+^) (**Figure 4D**) and CD8^+^ (CD45RA^-^ CD27^+^ CCR7^+^ CD3^+^ CD8^+^) (**Figure 4E**) (*p* ≤ 0.05) circulating central memory T cells (TCM) in the vaccinated volunteers from day 1 to day 28. Similarly, there is an increase in CD69^+^ T cells (CD45^+^ CD3^+^ CD69^+^) in the PBMCs following S-protein stimulation (*p* ≤ 0.05) (**Figure 4G**).

Additionally, there is a significant increase in the percentage of S-protein specific B cells in the PBMCs by 2 months post-initial injection (*p* ≤ 0.01) (**Figure 4H**) and a similar trend was also observed in S-protein specific memory B cells (*p* ≤ 0.01) (**Figure 4I**). B cell class switching was observed by 2 months post-initial injection, which saw a general trend of reduction in IgM^+^ memory B cell numbers (CD19^+^ CD27^+^) (**Figure 4J**) and an increase in IgG^+^ memory B cell numbers (CD19^+^ CD27^+^) (*p* ≤ 0.01) (**Figure 4K**). This corroborates the mouse data, demonstrating the presence of antigen specific memory B cells in vaccinated volunteers 28 days post-EDV-COVID-αGC vaccination, with seroconversion appearing to occur at around the 60 days post-initial injection mark.

## Discussion

Despite an unprecedented global effort to vaccinate against SARS-CoV-2, the cause of the COVID-19 pandemic, the continuous emergence of VOC has resulted in the decline of vaccine protective efficacy, necessitating reformulation of vaccines with dominant VOC S-proteins. Further, the currently approved vaccines have logistic issues such as the requirement to store and transport vaccines at -20°C to -70°C and a shelf life of only 3 to 12 months, making it difficult to deploy these vaccines to rural populations, especially in less developed regions of the world. There is an urgent need for a vaccine which protects against multiple VOCs, has a B- and T-cell memory, obviating the need for multiple booster doses, and which could be stored and transported at room temperature.

Here we provide data on a novel COVID-19 vaccine that readily overcomes these limitations.

The vaccine comprises a 400 nm non-living, achromosomal nanocell (EDV; EnGeneIC Dream Vector) derived from a non-pathogenic strain of *Salmonella enterica* serovar Typhimurium. The purified EDVs are pre-packaged with a bacterial gene expression recombinant plasmid carrying the SARS-CoV-2 S-protein encoding gene under a constitutive gene expression, modified β-lactamase promoter (EDV-COVID). The plasmid expresses the S-protein in the bacterial cytoplasm during normal bacterial growth and when the EDV is formed, a significant concentration of the S-protein segregates into the EDV cytoplasm. Additionally, the EDV-COVID nanocells are further packaged with αGC (EDV-COVID-αGC).

In previous studies we tracked the uptake and intracellular fate of the nanocells carrying different payloads being cytotoxic drugs (14) or nucleic acids (15). These studies, carried out in professional phagocytic cells (macrophages and dendritic cells) as well as non-phagocytic cells including various tumor cell lines, showed that cells rapidly phagocytose the nanocells and internalize them into early endosomes which then fuse into intracellular lysosomes and it is in these organelles that the nanocells are degraded presumably *via* the strong acidic environment and proteolytic enzymes. The payload is released in the lysosomes and it rapidly diffuses into the cellular cytoplasm of the cell (25). Flow cytometry studies showed that EDV-COVID-αGC effectively delivered both S-polypeptides and αGC into murine bone marrow derived JAWSII DCs. This is the first report that a single DC can present two different classes of molecule on its surface at the same time, where αGC was presented on the DC surface through glycolipid antigen presenting MHC Class I-like molecule, CD1d and S-polypeptides were presented likely *via* cell-surface MHC Class II molecules.

In vaccines comprising bacterial and viral vectors, immune responses that are specific for the corresponding bacterial or viral pathogen are induced when the vaccine vectors are live attenuated bacterial or viral vectors (4).

In contrast, the EDV, while bacterially-derived, is a non-living nanocell and does not drive the same immune responses as a live pathogen. These nanocells can therefore be constructed to elicit the desired immune response including expression of S-protein to stimulate anti-SARS-CoV-2 antibodies, inclusion of α-GC to direct the iNKT/DC/T_FH_ cell licensing to enhance the antibody affinity to neutralize the variety of mutant S-protein RBDs, and inclusion of plasmid to provoke a non-specific anti-viral IFNα response. Pathogen-associated molecular patterns (PAMPs) associated with the EDV such as LPS, provoke a mild IL-6 and TNFα response, and both these cytokines are conducive to an anti-viral response and do not stimulate neutralizing antibodies.

**Figure 5:**
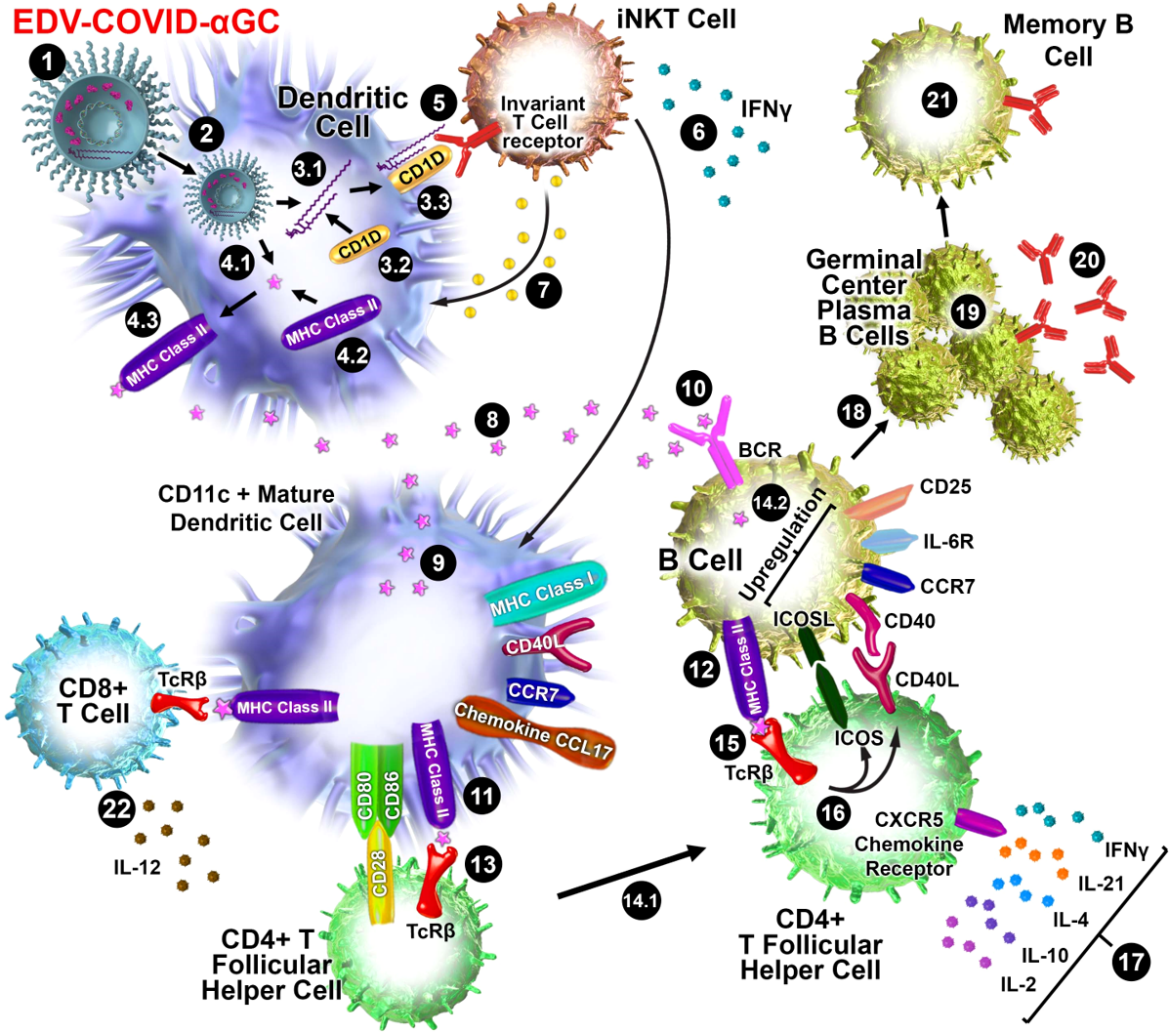
Schematic diagram of iNKT-licensed dendritic cell activation (1). EDV-COVID-αGC injected i.m. in mice (2), phagocytosed by dendritic cells (DC), degraded in lysosomes, (3.1) αGC released from EDVs, (3.2) CD1d binds to αGC, (3.3) CD1d:αGC complex displayed on DC cell surface, (4.1) spike polypeptides also released from EDVs, (4.2) MHC Class II binds to the S-peptides, (4.3) display them on same DC cell surface (5). iNKT semi-invariant T cell receptor binds to CD1d/αGC complex (6), rapidly secretes IFNγ which triggers upregulation of CD40 ligand in DCs inducing DC maturation/activation with increased costimulatory capacity through upregulation of CD80, CD86, CCR7, MHC Class I molecules, pro-inflammatory cytokine IL-12 & chemokine CCL17 (7). Binding of CD1d:αGC complex to the iNKT TCR triggers perforin release which kills the CD1d/αGC complex displaying DCs (8). S-polypeptides are released from dying DCs (9), endocytosed by activated CD11c+ DCs and (10) naïve B cells *via* B cell surface receptor, and (11, 12) displayed on each cell surface *via* MHC Class II (13). MHC Class II/spike on DC surface binds to CD4+ TCRβ on CD4+ T follicular helper (T_FH_) cells, and (14.1) these signals induce the T_FH_ cell differentiation and upregulation of chemokine receptor CXCR5 and downregulation of CCR7, which allows these cells to migrate to the T/B border. (14.2) B cells activated by S-polypeptide engagement of BCR increase CCR7 expression and migrates to the T/B follicle border in search of cognate CD4+ T cells (15). Recognition of the S-peptide/MHC II complex on B cells by the TCRβ enables T_FH_ cells (16) to express CD40 ligand and ICOS and (17) secretes the cytokines IL-21, IFNγ, IL-4, IL-2, and IL-10. T_FH_ cells are strongly enriched for cells expressing the highest levels of IL-21 (18). this cognate help stimulates B cells to undergo intense proliferation, induction of Ig class switching, differentiation to plasma-like cells capable of secreting all major Ig isotypes (19). Within GCs, B cells undergo somatic hypermutation and only B cells with the highest affinity antibody are selected (20). These plasma cells secrete high affinity S-specific antibodies that can neutralize a variety of S-mutants (21). These B cells differentiate into long-lived memory B cells. Throughout this process, IL-21 induces expression of CD25, enabling the B cells to respond to IL-2, also derived from T_FH_ cells, which promotes the effect of IL-21. Similarly, IL-21 induces expression of IL-6R on PCs, which allows these cells to integrate survival signals by IL-6 (22). DCs displaying S-peptides *via* MHC class II also elicit an S-specific CD8+ T cell response.

The display of αGC:CD1d on the DC cell surface recruits iNKT cells which carry the invariant TCR that is known to bind to CD1d-associated αGC on DCs, resulting in rapid secretion of IFNγ (26) as seen in only the EDV-COVID-αGC group of mice (**Figure 1G**). Vaccination using EDV-COVID-αGC resulted in significant serum IFNγ release by day 28 in the 6 human volunteers presented here (**Figure 4B**), suggesting activation of iNKT cells *via* the αGC:CD1d display on APCs (**Figure 5**). In contrast the currently approved mRNA vaccine (BNT162b2) showed transient serum IFNγ release, which wanes by day 8 (27). This is not surprising since the mRNA vaccines do not elicit antigen-specific antibodies *via* the iNKT/DC pathway. This iNKT cell activation and IFNγ secretion is critical in the activation of the high-affinity antibody production pathway depicted in **Figure 5**.

It has been demonstrated that activated iNKT cells promote DC maturation *via* CD40/40L signaling and cytokines IFNγ and TNFα (28). Additionally, the DCs engulfing the EDVs are further activated *via* the pathogen-associated molecular patterns (PAMPs) like EDV-associated LPS (25). This activation releases TNFα which is evident in all four EDV containing groups (**Figure 1J**). DC maturation leads to secretion of IL-12, a loop feedback iNKT induction cytokine, only observed here in the EDV-COVID-αGC group (**Figure 1H**) providing evidence of iNKT pathway engagement. This promotes the cytolytic function of cytotoxic CD8^+^ T cells and priming of CD4^+^ T cells (29) to provide cognate help to B cells for antibody production. In our mouse studies we observed higher IFNα and IL-12p40 cytokine levels in the 2 x 10^9^ particle dose versus 3 x 10^9^. This could be the result of a different level of response at the chosen time of sampling as a consequence of different doses (30).

The data presented here suggests that EDVs carrying S-protein were able to induce CD4^+^ and CD8^+^ T cell specificity and memory in mice and humans, further enhanced by the inclusion of αGC. Overall, our T cell analyses indicate that the population of circulating T cells resulting from EDV-COVID-αGC vaccination of mice and human volunteers, were SARS-CoV-2 S-protein specific, as observed with other vaccines (31).

It has been established that B cells that have MHC Class II presented protein antigen first engage in cognate interactions with T_FH_ cells at the junction between the T cell–rich areas and B cell follicles of secondary lymphoid tissues (32–34). Engagement of MHC Class II/antigen complex on these B cells with the T_FH_ cell surface TCR results in the rapid upregulation of cognate helper co-stimulatory molecules CD40L (35), inducible T cell co-stimulator ICOS (36) and PD-1 (**Figure 5**).

Binding of ICOS ligand, which is expressed on naive B cells (37), to ICOS on T_FH_ cells is essential for the progression of pre-T_FH_ to fully differentiated T_FH_ cells. ICOS/ICOSL signalling also leads to the release of multiple cytokines including IFNγ, IL‐4, IL‐10, IL‐17, IL‐2, IL-6 and IL-21 (38–41).

Splenocytes from mice immunised with EDVs when stimulated with S-protein trimer showed that CD4^+^ T cells from EDV-COVID and EDV-COVID-αGC mice, but not those from other groups, produced IFNγ but not IL-4 (**Figure 3E**) indicating that CD4^+^ T cells were primed to elicit an antigen specific Th1 type response following vaccination.

B cell activation results in either the extrafollicular proliferation of long-lived antibody producing B cells as plasmablasts or their entry into GCs for the subsequent development of memory or plasma cells (42). Post-T_FH_ cell cognate interactions in the follicles, proliferating B cells give rise to GCs and undergo somatic hypermutation (SHM) in their immunoglobulin V-region genes and affinity maturation which produces plasma and memory cells of higher affinity (43–49). Within the GC, T_FH_ cells provide further B cell help mainly through the secretion of IL-21 and CD40L co-stimulation, which are two major factors for B cell activation and differentiation. IL-21 also induces class switching to IgG1 and IgG3 by human naive B cells and increased secretion of these Ig isotypes by human memory B cells (50).

IL-21 is mainly expressed by T_FH_ cells and stimulates the proliferation of B cells and their differentiation into plasma cells. Class switching to IgG and IgA of CD40L-interacting B cells is also promoted by IL-21 (51). A highly significant increase in IL-21 was observed only in EDV-COVID-αGC treated mice (**Figure 1L**) demonstrating the addition of αGC was essential in activating CD4^+^ T_FH_ cells likely due to iNKT-licensed DC activation of T_FH_ cells as described above. For development of S-specific antibody responses, IL-21 plays a critical role in T cell–dependent B cell activation, differentiation, germinal center (GC) reactions (52) and selection of B cells secreting antigen-specific high affinity antibodies.

B cells isolated from mouse bone marrow at 28-day post-initial injection when co-incubated with S-protein showed that B cells from EDV-COVID-αGC immunized mice produced significantly higher levels of S-specific IgM (**Figure 3A**) and IgG (**Figure 3B**) compared to all other groups. This indicates that EDV-COVID-αGC treatment induced SARS-CoV-2 specific memory B cells that could respond rapidly to S-protein re-exposure.

In a similar fashion, the presence of antigen specific memory B cells was detected in the PBMCs of the 6 healthy volunteers 28 days post-EDV-COVID-αGC vaccination (**Figure 4I**), with seroconversion appearing to occur at around the 60 days post-initial injection (**Figures 4J, K**).

At both dose levels, high levels of anti-S protein IgM (**Figure 2A**) and IgG (**Figure 2B**) antibody titers were detected in the serum of most mice immunized with EDV-COVID-αGC at 28 days post-initial dose and a booster dose at day 21. IgM and IgG antibodies were also elicited by mice immunized with EDV-COVID but the IgG response was lower (**Figures 2A, B**). The inclusion of αGC into EDV-COVID resulted in a dramatic and consistent elevation of S-specific IgG titers. S-specific IgG responses by days 7 and 21 with EDV-COVID and EDV-COVID-αGC were similar **(Figures 2D, F**) however, the booster effect was pronounced on day 28 only in EDV-COVID-αGC immunized mice showing that the incorporation of αGC into EDV-COVID led to an iNKT-licensed DC pathway, known to result in germinal center B cell activation/maturation with high titer antibody secretion. The surrogate virus neutralization analyses in mice are indicative of this, since the addition of αGC neutralized VOC tested here more effectively.

Serum sVNT from 6 healthy volunteers from our Covid vaccine phase I clinical trial exhibited strong neutralizing activity (>PRNT_90_ equivalent) against ancestral, Delta and Omicron B.1.1.529 by day 28 (**Figure 4Ai**). Neutralizing activity was present for Omicron B.1.1.529 and BA. 2 as well at 3 months compared to B.1.1.529 data from 5 volunteers who had received 2 doses of BNT262b2 3-4 months previously (**Figure 4Aii**).

In addition, we found the neutralization level against BA. 4/5 increased significantly in volunteers who had previously received other available vaccines and were then boosted with EDV-COVID-αGC at least 4 months later (**Figure 4Aii**). The human trial data is consistent with the mouse results suggesting that the EDV-COVID-αGC vaccine elicits high affinity antibodies that are able to effectively neutralize the ancestral as well as Omicron VOC RBDs.

In contrast, the data for the mRNA vaccine (BNT162b2) showed reductions of 2-fold (Alpha), 5 to 10-fold (Beta), 2 to 5-fold (Gamma), 2 to 10-fold (Delta), to achieve PRNT_50_ (53) and over 22-fold for Omicron in neutralization efficiencies (54). The current spike in number of infections around the world despite high vaccination rates, along with the continued requirement for booster shots reflects the reduction in neutralization efficiencies of the currently available vaccines against the dominant Omicron variants.

Further studies are necessary to determine the nature of the difference in antibodies that are generated by EDV-COVID and EDV-COVID-αGC vaccines and why antibodies elicited by the latter are significantly more effective at neutralization of various VOC RBDs. Quantitative studies on antibody affinity/avidity as well as RBD epitope characterization should assist in answering these questions.

It is currently thought that successful vaccination relies on both antibody- and T cell-mediated immunity and while it is recognized that at least Type I and Type II interferons can elicit a broad anti-viral immunity, due to the multitude of effects that these interferons exhibit, it is quite possible that to curb the current and future viral pandemics, a broad specific and non-specific anti-viral immunity combined with a specific memory B and T cell response is necessary.

EDV based cancer therapeutics or COVID vaccines are lyophilized post-manufacturing and can be stored and transported world-wide at room temperature. The shelf life of EDV cancer therapeutics have currently been shown to be over 3 years and the EDV-COVID-αGC vaccine has exceeded 1 year of stability.

## Data availability statement

The original contributions presented in the study are included in the article/supplementary material. Further inquiries can be directed to the corresponding author.

## Ethics statement

The studies involving human participants were reviewed and approved by St. Vincent’s Hospital Melbourne Human Research Ethics Committee. The patients/participants provided their written informed consent to participate in this study. The animal study was reviewed and approved by EnGeneIC Animal Ethics Committee.

## Author contributions

HB, JM conceptualized the study. HB, JM, SG, NA-M designed the study. SG, NA-M supervised all the experiments. SG, NA-M, JM-W, NP, NV performed the experiments and collated all the data. JM-W led and supervised mouse experiments. BW helped with discussions and critical review of the manuscript. HB, JM, SG, and NA-M wrote the manuscript with input from all the authors. KV was the principal investigator in the EDV-COVID Phase I clinical trial. All authors contributed to the article and approved the submitted version.
