# Supplementary material for: Nanocell COVID-19 vaccine triggers a novel immune response pathway producing high-affinity antibodies which neutralize all variants of concern
Source: Front Immunol. 2023 Jan 27;13:1038562. doi: 10.3389/fimmu.2022.1038562 (PMC9929940; doi:10.3389/fimmu.2022.1038562)
Supplement: Supplementary file 1 [file DataSheet_1.docx]

Supplementary Materials for

Nanocell COVID-19 vaccine elicits iNKT-licensed dendritic cells to produce high affinity antibodies neutralizing Variants of Concern

Steven Y. Gao^1†^, Nancy B. Amaro-Mugridge^1†^, Jocelyn Madrid-Weiss^1^, Nikolina Petkovic^1^, Natasha Vanegas^1^, Kumar Visvanathan^2^, Bryan R. G. Williams^3^, Jennifer A. MacDiarmid^1¥^, Himanshu Brahmbhatt^1¥*^

*Corresponding author. Email: hbrahmbhatt@engeneic.com

**This PDF file includes:**

Figs. S1 to S3

Table S1-S3


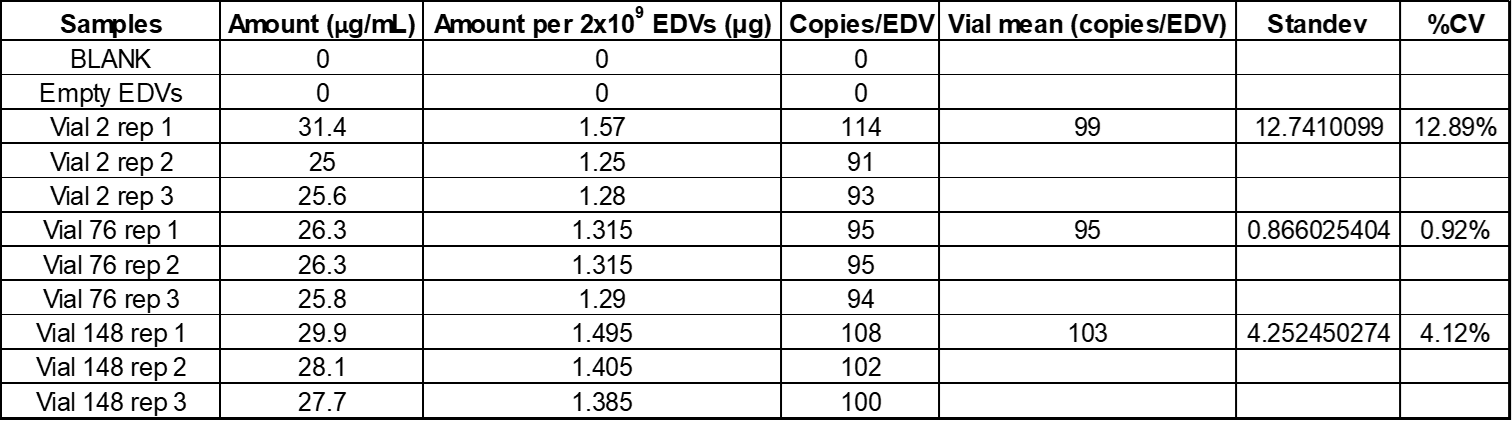


**Table S1.** An example of plasmid copy number analysis of a batch of EDV-COVID-αGC.

| **ELISA Antibodies** |  | |
| --- | --- | --- |
| Mouse TNFα | DuoSet R&D Systems | DY41005 |
| Mouse IFNαall subtype | Verikine-HS PBL | 42115 |
| Mouse IFNγ | DuoSet R&D Systems | DY48505 |
| Mouse IL12/p40 | DuoSet R&D Systems | DY239805 |
| Mouse IL4 | DuoSet R&D Systems | DY40405 |
| Mouse IL10 | DuoSet R&D Systems | DY41705 |
| Mouse IL6 | DuoSet R&D Systems | DY40605 |
| Mouse IL2 | DuoSet R&D Systems | DY402 |
| Mouse IL21 | LEGEND MAX Mouse IL-21 ELISA Kit (Biolegend) | 446107 |

**Table S2.** ELISA kits used for cytokine analysis.

| **Marker** | **Antibody** | **Quantity**  **µg/500µl/1x10^6^ cells** | **Manufacturer** |
| --- | --- | --- | --- |
| CD45 | Anti-mouse CD45-AF488 | 1.5 µg | Biolegend 103122 |
| CD4 | Anti-mouse CD4 [GK1.5] (PE-Texas Red®) | 0.15 µg | Abcam ab51467 |
| OX40 | Anti-mouse CD134 (OX-40)-APC | 0.3 µg | Biolegend 119413 |
| CD3 | Anti-mouse CD3-PE-Cy7 | 0.75 µg | Biolegend 100220 |
| CD8 | Anti-human/mouse CD8-AF647 | 0.75 µg | Biolegend 372906 |
| CD69 | Anti-mouse CD69-BV421 | 0.075 µg | Biolegend 104527 |
| 7-AAD | Viability Staining Solution | 4 µl | Biolegend 420404 |

**Table S3.** T cell staining panel.


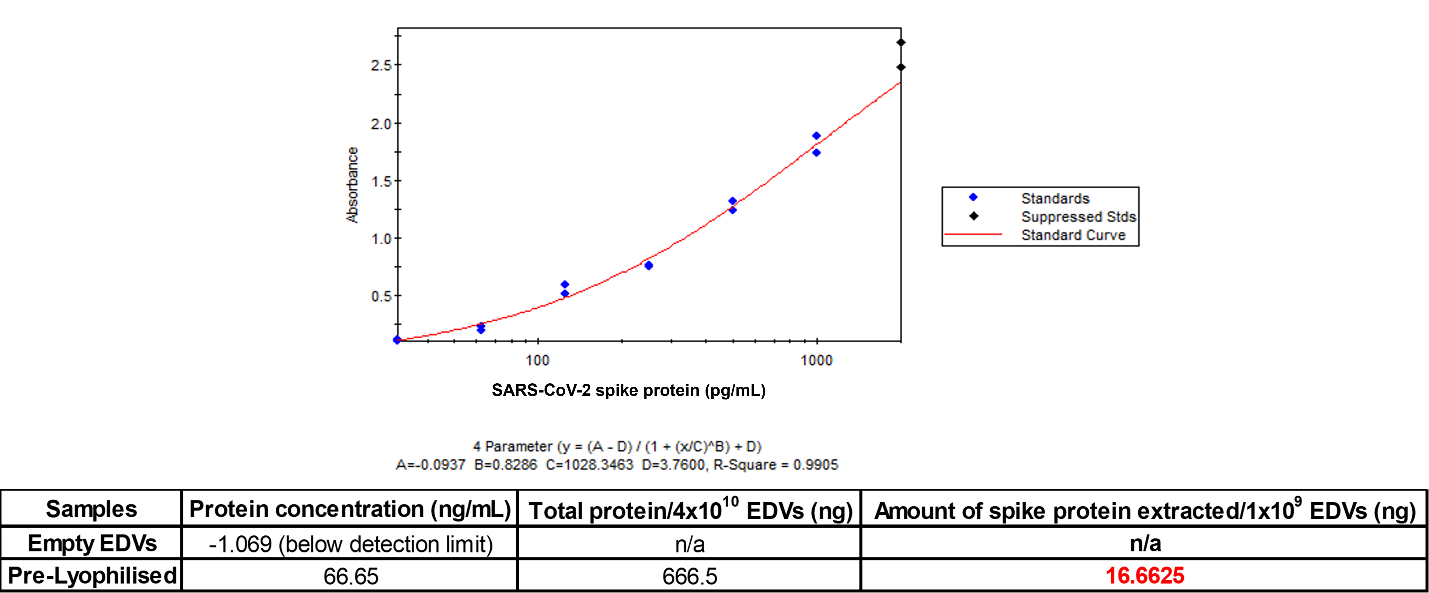


**Figure. S1.** SARS-CoV-2 spike protein quantification using ELISA.


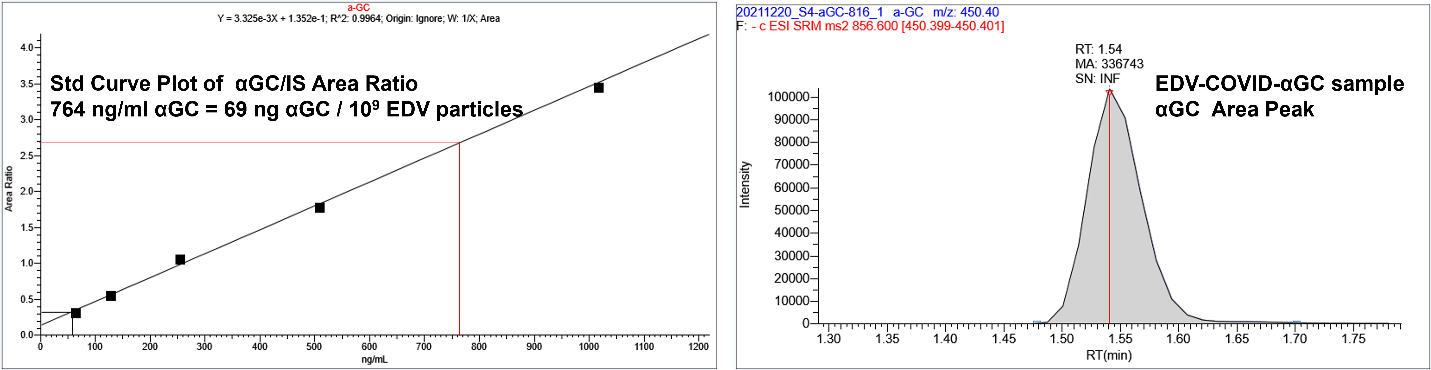


**Figure. S2.** LC-MS αGC quantitation standard curve and area under the peak for EDV-COVID-αGC extracted particles.


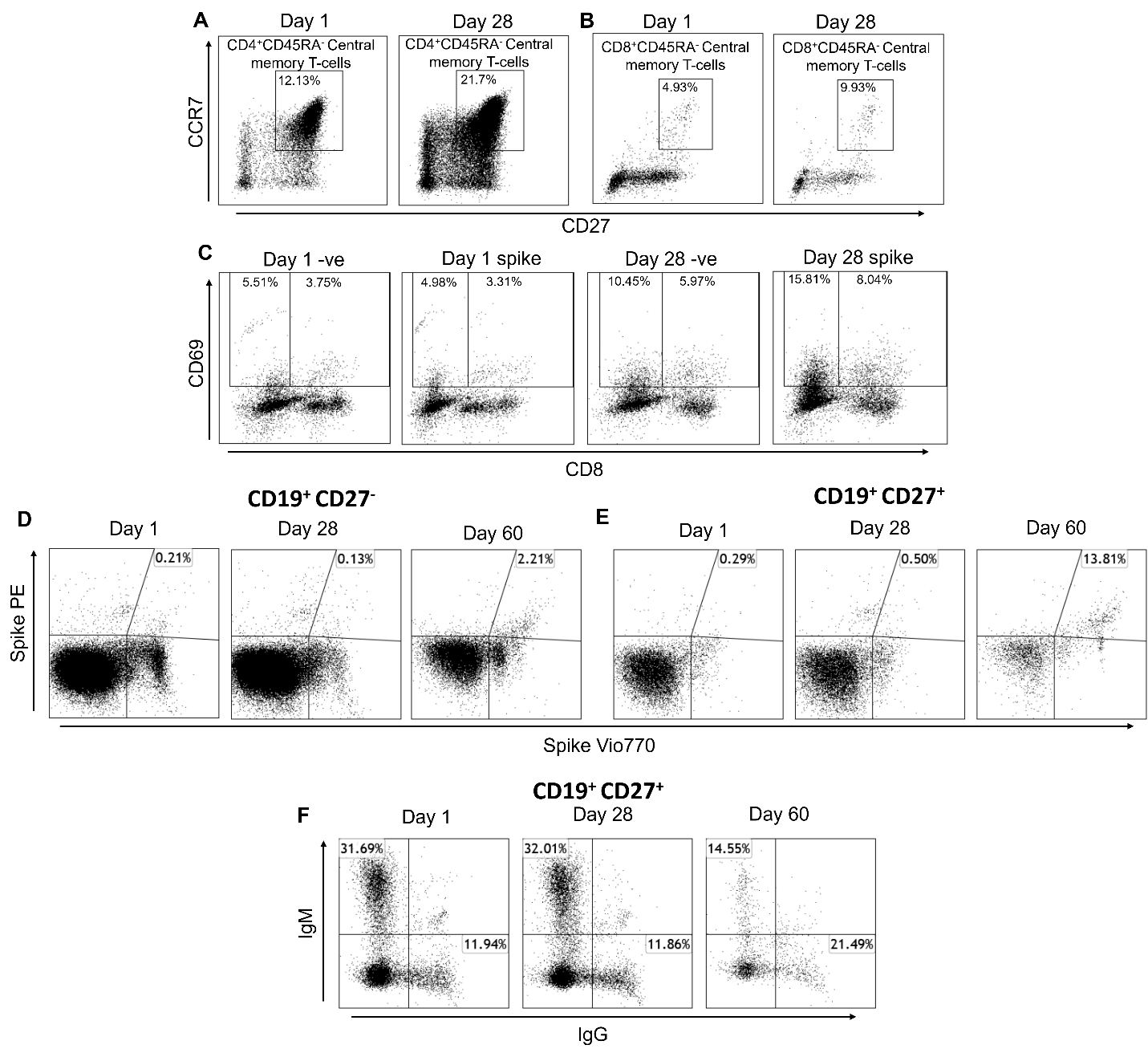


**Figure. S3.** Represented images of FACS analysis of human trial samples. (A) CD4^+^ central memory T cells from PBMCs isolated on day 1 and day 28 post-initial injection. (B) CD8^+^ central memory T cells from PBMCs isolated on day 1 and day 28 post-initial injection. (C) CD69+ T cell expression from ex-vivo stimulated PBMCs isolated on day 1 and day 28 post-initial vaccination. –ve: stimulated with DMSO, spike: stimulated with SRAS-CoV2 spike protein. (D) Analysis of SARS-CoV2 spike protein specific B cells on day 1, day 28 and day 60 post-initial injection. The spike protein specificity was determined by double labelling the B cells with anti-spike Vio770 and anti-spike PE. (E) Analysis of SARS-CoV2 spike protein specific CD19^+^ CD27^+^ memory B cells on day 1, day 28 and day 60 post-initial injection. The spike protein specificity was determined by double labelling the B cells with anti-spike Vio770 and anti-spike PE. (F) Representative images of IgM^+^ and IgG^+^ CD19^+^ CD27^+^ memory B cells on day 1, day 28 and day 60 post-initial injection.
